# Racial and Ethnic Disparities in Cost-Related Healthcare Access Barriers in Florida (2012–2024): A Joinpoint Regression and Bayesian Analysis

**DOI:** 10.7759/cureus.105645

**Published:** 2026-03-22

**Authors:** Carlos Acosta-Batista, Berenice D Pena Reina, Jeniffer S Romero, Loraine Gonzalez Chirino, Antoinette Contino Rivero, Nathali Gonzalez Reyes

**Affiliations:** 1 Clinical Research Department, Primary Care - Research Initiative (PCRI), Miami Lakes, USA

**Keywords:** access to healthcare, bayesian inference, disparities in healthcare, healthcare access barriers, public health policy

## Abstract

Introduction: Economic barriers to healthcare disproportionately affect racial and ethnic minorities. While the Affordable Care Act (ACA) aimed to reduce these disparities, the COVID-19 pandemic represented an unprecedented external shock. This study aims to quantify state-level trends in cost-related healthcare access barriers in Florida, focusing on intersectional vulnerabilities.

Methods: We conducted an ecological time-series analysis using aggregated state-level data (*N *= 78 total data points) from the Behavioral Risk Factor Surveillance System (BRFSS) for Florida adults (2012-2024). The primary outcome was the annual prevalence of adults unable to see a doctor due to cost, stratified by sex and ethnicity (Hispanic, non-Hispanic Black, and non-Hispanic White). Bayesian paired samples analysis and frequentist inference were used to estimate structural disparities, and restricted piecewise linear regression (Joinpoint) evaluated temporal trend reversals.

Results: Over the 13-year period, Hispanic women exhibited the highest temporal average barrier rate (23.81%). Bayesian paired inference demonstrated very strong evidence (Bayes factor_10_ = 7.18 x 10^4^) of a structural gap, with Hispanic women facing a mean paired difference 13.02 percentage points higher than that of non-Hispanic White men (95% confidence interval of the paired difference: 10.29-15.75; *p* < 0.001). Trend analysis revealed a critical structural break for Hispanic women in 2020: prior to the pandemic, barriers were declining at an absolute annual change (AAC) of -2.06 (*p* = 0.001); post-2020, this protective trend was completely halted, shifting into an upward trajectory (+1.28 AAC, *p* = 0.327).

Conclusions: State-level macro-trends demonstrate that the onset of the COVID-19 pandemic coincided with a pronounced structural break, abruptly halting previous gains in healthcare access for the Hispanic female cohort in Florida. These ecological findings underscore the profound intersectional vulnerability of the primary care safety net during periods of systemic disruption.

## Introduction

Cost-related healthcare barriers disproportionately impact racial and ethnic minority groups, reinforcing historical inequalities in health service access [1,2]. While Hispanic individuals constitute the largest minority population in the United States, treating them as a homogeneous entity masks substantial heterogeneity in socioeconomic status, insurance coverage, and health outcomes [3,4]. Florida presents a critical setting to examine these disparities due to its large Hispanic population and its status as a Medicaid non-expansion state under the Affordable Care Act (ACA), conditions that exacerbate structural inequalities. Although the ACA successfully reduced uninsured rates, evidence suggests that expanding insurance coverage alone does not eliminate financial barriers to care, particularly among minority populations [5]. Furthermore, while the COVID-19 pandemic severely altered economic conditions and social determinants of health, recent ecological evidence indicates that the Hispanic paradox persists regarding mortality in Florida [6].

Despite the extensive literature on health disparities, critical knowledge gaps remain regarding the intersectionality of race, ethnicity, and sex over time. Many existing studies rely on cross-sectional designs that cannot quantify the persistence of disparities amidst major exogenous shocks. Therefore, the primary objective of this ecological brief report is to examine temporal macro-trends in cost-related healthcare access barriers in Florida (2012-2024) among Hispanic, non-Hispanic White, and non-Hispanic Black adult cohorts, explicitly stratified by sex. We aim to identify critical structural trend reversals associated with the onset of the COVID-19 pandemic. In addition, Bayesian modeling is employed to formally quantify the magnitude and certainty of the ecological intersectional disparity, evaluating the absolute state-level equity gap in primary care access across the study period.

## Materials and methods

Study design and data source

An ecological, macro-level time-series analysis was conducted using aggregated annual cross-sectional data from 2012 to 2024. Data were extracted from the Behavioral Risk Factor Surveillance System (BRFSS) web portal for the state of Florida, maintained in collaboration with the Centers for Disease Control and Prevention (CDC) [7]. As the dataset is publicly accessible and consists of fully de-identified surveillance data, no data use permission or institutional license was required for access. Rather than analyzing individual-level microdata, this study utilized state-level prevalence aggregates as the primary units of analysis, yielding a total of 78 data points (13 annual estimates across six demographic cohorts). This ecological approach was specifically chosen to evaluate state-level macro-trends and the systemic behavioral responses to major public health and policy events.

Variables and cohorts

The primary outcome was the aggregated annual prevalence of cost-related healthcare access barriers, defined by the self-reported inability to see a doctor due to cost in the past 12 months. The data were extracted as pre-aggregated estimates directly from the Florida Department of Health’s FLHealthCHARTS portal [7]. Therefore, demographic categories were operationalized according to the standardized reporting methodology of the Florida BRFSS. The data were stratified into six distinct, state-level demographic cohorts based on mutually exclusive race/ethnicity and sex categories: (1) Hispanic women, (2) Hispanic men, (3) non-Hispanic Black women, (4) non-Hispanic Black men, (5) non-Hispanic White women, and (6) non-Hispanic White men (reference group). Because this study utilized pre-aggregated ecological data to assess macro-trends, individual-level demographic characteristics (e.g., mean age, household income, or education level) were inherently inaccessible and thus not included in the analysis. Under the Florida Department of Health's reporting framework, individuals identifying as multiracial or those with missing, "don't know", or refused responses for these demographic fields are structurally excluded from the denominators of these specific cohort estimates, ensuring longitudinal homogeneity for the comparison groups.

Statistical analysis

Descriptive statistics, including temporal means and standard deviations (SD), were calculated for each demographic cohort to summarize the aggregate longitudinal variance of the access barrier prevalence across the 13-year study period.

To account for temporal autocorrelation and shared state-level macroeconomic shocks across the decade, structural disparities were evaluated using a Bayesian paired samples *t*-test. By matching the aggregated prevalence rates year-by-year (*N*=13 pairs per comparison), this approach isolates the underlying structural "equity gap" from the longitudinal variance of the time series. Rather than employing strictly non-informative priors, which can artificially induce support for the null hypothesis in model selection (the Jeffreys-Lindley paradox), a default objective Cauchy prior distribution (scale *r* = 0.707) centered on zero was specified for the effect size [8]. This approach provides heavy tails to realistically accommodate potential structural differences without diluting the prior probability mass toward infinity. The resulting Bayes factor (*BF*_10_) was interpreted according to the classification framework proposed by Kass and Raftery, where a *BF*_10_ > 150 indicates very strong evidence in favor of the alternative hypothesis [9].

Concurrently, a frequentist paired Student’s *t*-test was utilized to ensure methodological robustness across both inferential frameworks. For full transparency, all frequentist test statistics, including the *t*-value and degrees of freedom (*df*), are reported alongside the 95% confidence intervals (CIs) of the mean paired differences and their corresponding exact *p*-values.

To evaluate temporal trend reversals, a restricted piecewise linear regression (Joinpoint) was employed. To ensure parsimony and prevent mathematical overfitting, the algorithm was constrained to identify a maximum of one structural inflection point. The optimal number of joinpoints (0 vs. 1) and the exact location of the knot were not fixed a priori; rather, they were determined empirically using the weighted Bayesian information criterion (WBIC). The rate of trend progression is reported as the absolute annual change (AAC) in percentage points, accurately reflecting absolute state-level variance rather than log-linear exponential rates. Furthermore, to mathematically quantify the magnitude of any detected structural break, a parameterized *t-*test was utilized to evaluate the statistical significance of the difference between the pre- and post-inflection slopes. Statistical significance was established at an alpha level of *p* < 0.05. For all Joinpoint trend segments, the corresponding *t*-value, *df*, and *p*-value are comprehensively reported.

Software

Data cleaning, descriptive statistics, and Bayesian inference were performed using Python (v3.10+). Specific statistical procedures, including frequentist and Bayesian paired samples *t*-tests, as well as posterior probability density estimations, utilized the SciPy and Statsmodels libraries. The temporal trend analysis and empirical structural break detection (inflection points) were executed using the Joinpoint Regression Program, version 5.2.0 (Statistical Methodology and Applications Branch, National Cancer Institute, MD, USA).

Ethics statement

This study utilized publicly available, fully de-identified, and aggregated data from the BRFSS. Therefore, it was exempt from Institutional Review Board (IRB) approval.

## Results

Baseline structural and intersectional disparities

Between 2012 and 2024, the state-level prevalence of cost-related healthcare barriers in Florida was highly stratified by sex and ethnicity (Table 1). Hispanic women experienced the highest mean barrier rate across the decade (23.81%, SD ± 5.72), while non-Hispanic White men consistently reported the lowest barriers (10.78%, SD ± 2.24). 

**Table 1:** Descriptive prevalence and paired structural disparities (equity gap) in cost-related barriers in Florida (2012–2024) Note: Values represent the aggregated state-level percentage of adults reporting an inability to see a doctor in the past 12 months due to cost. Data sourced from the Behavioral Risk Factor Surveillance System (BRFSS). The 95% confidence interval (CI) reflects the temporal variation of the state-level estimates across the 13-year study period. The "temporal mean" and "temporal SD" summarize the crude state-level descriptive prevalence and its longitudinal variance across the 13-year period (N = 13 annual estimates per cohort). To control for temporal autocorrelation, inferential statistics (mean paired difference and 95% CI) were calculated using year-over-year matched pairs relative to the non-Hispanic White male reference group. Bayes factors (BF10) were interpreted according to the Kass and Raftery scale, where values >150 indicate very strong evidence of an underlying structural gap independent of longitudinal trend variance. SD: standard deviation

Demographic group	Temporal mean (%) + SD	Mean paired difference (%) + SD	95% CI of the difference	Bayes factor (*BF*_10_​)
Hispanic women	23.81 ± 5.72	+ 13.02 ± 4.52	10.29–15.75	7.18 x 10^4^
Non-Hispanic Black women	21.02 ± 5.63	+ 10.24 ± 4.21	7.69–12.78	1.32 x 10^4^
Hispanic men	20.02 ± 3.50	+ 9.23 ± 2.57	7.68–10.79	6.81 x 10^5^
Non-Hispanic Black men	16.45 ± 3.75	+ 5.66 ± 2.61	4.09–7.24	4.40 x 10^3^
Non-Hispanic White women	14.01 ± 2.35	+ 3.22 ± 1.27	2.45–3.99	1.98 x 10^4^
Non-Hispanic White men	10.78 ± 2.24	Reference	-	-

Bayesian paired samples analysis showed a *BF*_10_ = 7.18 x 10^4^, demonstrating very strong evidence (*BF*_10_ > 150) of a persistent structural gap. By matching the data year-by-year to control for shared temporal trends, the analysis revealed that Hispanic women faced an annual cost-barrier prevalence that was, on average, 13.02 percentage points higher than the non-Hispanic White male reference group (95% CI of the paired difference: 10.29 - 15.75; *t*(12) = 10.39, *p* < 0.001).

Furthermore, to isolate the specific ecological ethnic disparity independently of sex, Hispanic women were compared to non-Hispanic White women. This secondary paired decomposition also revealed very strong evidence (*BF*_10_ = 6.34 x 10^3^) of an enduring ethnic disparity, with Hispanic women experiencing a barrier prevalence 9.8 percentage points higher than their White female counterparts (95% CI of the paired difference: 7.17-12.43; *t*(12) = 8.13, *p* < 0.001).

**Figure 1:**
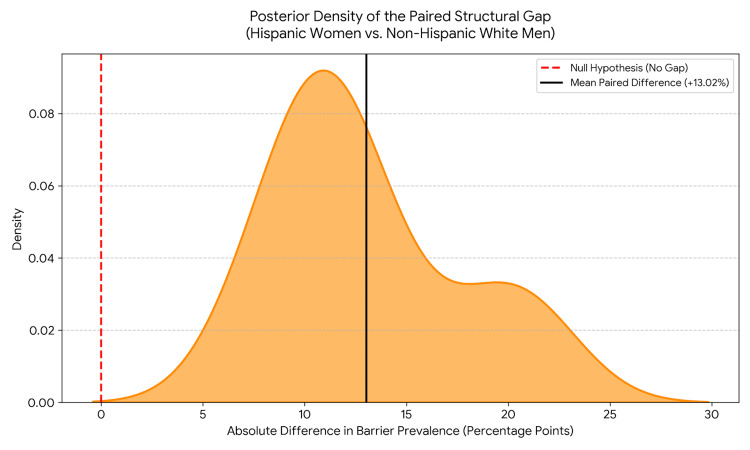
Bayesian posterior probability density of the paired structural gap. The density plot illustrates the distribution of the absolute paired difference in cost-related healthcare access barriers between Hispanic women and the Non-Hispanic White male reference group in Florida (2012–2024). The entire distribution of the year-over-year matched differences (orange shaded region) is shifted significantly to the right of the null hypothesis (red dashed line at 0 gap), clustering tightly around the mean paired difference of +13.02 percentage points (solid black line). Data sourced from the Behavioral Risk Factor Surveillance System (BRFSS).

Visual analysis of the posterior density for the paired difference (Figure 1) revealed a bimodal distribution, reflecting two distinct temporal magnitudes of the structural gap. The smaller rightward peak captures the earliest years of the study period (2012-2014), characterized by severe intersectional disparities where the paired difference peaked between 18.8 and 21.7 percentage points. The larger leftward peak represents the subsequent years (post-2014), where the absolute gap narrowed to approximately 10 to 12 percentage points. Crucially, the entire density distribution remained strictly bounded far above the null hypothesis of zero, underscoring that despite relative temporal improvements, intersectional equity was never achieved at any point during the 13-year period.

Temporal trends and pandemic reversal

**Figure 2:**
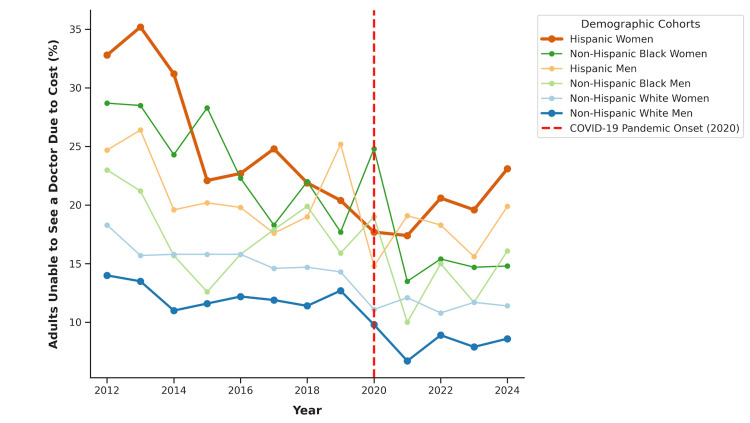
Ecological trends in cost-related healthcare access barriers in Florida, stratified by sex and ethnicity (2012–2024). The vertical dashed line (2020) marks the onset of the COVID-19 pandemic. Data sourced from the Behavioral Risk Factor Surveillance System (BRFSS).

Segmented piecewise linear regression, utilizing the WBIC for empirical model selection, identified a profound divergence in temporal trajectories. While baseline disparities were massive, the longitudinal impact of the COVID-19 macroeconomic shock was exclusively concentrated in a single demographic (Figure 2, Table 2).

The algorithmic model selection determined that Hispanic women were the only cohort to experience a statistically significant structural break during the study period (optimal model: 1 Joinpoint). Prior to 2020, state-level access barriers for Hispanic women were steadily improving, demonstrating a robust protective downward trend with an AAC of -2.06 percentage points *(t*(9) = -4.85, *p* = 0.001). However, the onset of the pandemic induced an acute structural reversal. Post-2020, this protective trajectory was completely halted, shifting into stagnation with an upward slope of +1.28 AAC (*t*(9) = 1.05, *p* = 0.327). The standard parameterization test confirmed that the magnitude of this structural break - the transition from a steep decline to an upward trajectory - was statistically significant (difference in slopes: +3.34; *t*(9) = 2.57, *p* = 0.033).

In stark contrast, the algorithm identified a 0-Joinpoint continuous model as the optimal fit for all other demographic cohorts. This indicates that these groups maintained their pre-existing trajectories without a significant structural fracture, converging with the global health crisis. Non-Hispanic Black women (AAC = -1.25; *t*(11) = -5.75, *p* < 0.001), non-Hispanic White women (AAC = -0.56; *t*(11) = -8.39, *p* < 0.001), and non-Hispanic White men (AAC = -0.48; *t*(11) = -5.08, *p* < 0.001) maintained continuous, statistically significant improvements across the entire observation period. Hispanic men (AAC = -0.51; *t*(11) = -2.32, *p* = 0.041) also exhibited a steady, uninterrupted decline. This underscores a unique intersectional vulnerability within the Hispanic female cohort, whose decade-long progress was the only one acutely and significantly dismantled by the systemic disruptions of 2020.

**Table 2:** Segmented trend analysis and empirical model selection (WBIC) of cost-related healthcare access barriers (2012–2024) The weighted Bayesian information criterion (WBIC) empirically determined the optimal number of structural breaks (Joinpoints). For 0-Joinpoint models, Segment 1 represents a single, continuous trajectory across the entire 13-year period, and Segment 2 is not applicable (—). Asterisks (*) indicate statistical significance at *p* < 0.05. AAC: absolute annual change in percentage points

Demographic cohort	Optimal model (WBIC)	Segment 1 years	Segment 1 AAC (*p*-value)	Segment 2 years	Segment 2 AAC (*p*-value)	Structural break
Hispanic women	1 Joinpoint	2012–2020	-2.06 (*p* = 0.001)*	2020–2024	+1.28 (*p* = 0.327)	*p* = 0.033*
Non-Hispanic Black women	0 Joinpoints	2012–2024	-1.25 (*p* < 0.001)*	—	—	No Break Detected
Non-Hispanic White women	0 Joinpoints	2012–2024	-0.56 (*p* < 0.001)*	—	—	No Break Detected
Non-Hispanic Black men	0 Joinpoints	2012–2024	-0.52 (*p* = 0.055)	—	—	No Break Detected
Hispanic men	0 Joinpoints	2012–2024	-0.51 (*p* = 0.041)*	—	—	No Break Detected
Non-Hispanic White men	0 Joinpoints	2012–2024	-0.48 (*p* < 0.001)*	—	—	No Break Detected

Furthermore, Table 2 indicates that while all female demographic cohorts exhibited statistically significant improvements in healthcare access prior to 2020 (all *p *< 0.05), none maintained a statistically significant trajectory of improvement during the post-pandemic period.

## Discussion

The analysis of BRFSS data from 2012 to 2024 revealed significant and structurally ingrained state-level disparities in cost-related healthcare access barriers across ethnic and sex cohorts in Florida. Bayesian paired samples analysis confirmed very strong evidence (*BF*_10_ = 7.18 x 10^4^) of a persistent macro-level gap, controlling for shared temporal trends and demonstrating that aggregate access barriers for Hispanic women remained vastly higher than those of the non-Hispanic White reference group throughout the decade. Furthermore, temporal trend analysis identified a distinct structural shift associated with the global health crisis. The transition from a robust and significant trajectory of improvement (AAC = -2.06; *p *= 0.001) to a phase of instability with an upward trend (AAC = +1.28; *p* = 0.327) following the 2020 systemic shock suggests a degradation of the healthcare access environment for Hispanic women. Although the post-pandemic period shows marginal significance due to the limited number of data points, the structural break identified by the Joinpoint model confirms a substantial deviation from the preceding decade of progress.

The identified 23.81% temporal mean barrier rate among Hispanic women in Florida represents a critical outlier. Recent federal surveillance data (2022 BRFSS) identifies the U.S. South Region as having the highest national prevalence of both health insurance gaps and cost-related barriers to medical care [10]. While Ayodele et al. established that Hispanic ethnicity and female sex serve as independent predictors of healthcare delays, our state-level analysis demonstrates that in Florida, the convergence of these factors creates a barrier magnitude that significantly exceeds national benchmarks [11]. This profound structural vulnerability aligns with the findings of Pedraza et al., who note that chronic financial challenges and health literacy barriers severely hampered the Latinx community's ability to seek timely care and treatment during the health crisis [12].

In evaluating the macro-trends of healthcare access, it is crucial to consider the broader policy landscape. Florida's status as a Medicaid non-expansion state has maintained a quantifiable coverage gap for low-income populations [13]; notably, citizen Latinos in low-income households in Florida had only a 23.9% probability of having any public health insurance, compared to 59.3% in New York, an expansion state [14]. This systemic lack of coverage is closely associated with persistent access barriers; Rivera-González et al. reported that 26.9% of Latinos in Florida experienced delayed care due to cost, showing only a marginal post-ACA improvement of -1.9% (95% CI, -3.1 to -0.8) between 2016 and 2019 [14]. Furthermore, these cost-related barriers frequently precipitate medical indebtedness, with 15.3% (95% CI, 14.4-16.2) of the uninsured carrying medical debt [15]. Acquiring such debt has been prospectively associated with exacerbating other social determinants of health, yielding an odds ratio (OR) of 2.20 (95% CI, 1.58-3.05) for becoming food insecure, and an OR of 2.29 (95% CI, 1.73-3.03) for losing the ability to pay rent or a mortgage [15]. Consequently, inadequate access to primary care is a well-documented driver of preventable emergency department (ED) visits among vulnerable populations [16]. In this setting, the implementation of state-level policies plays a critical role in mitigating disparities; for instance, the adoption of mandatory Medicaid managed care in Florida was associated with statistically significant reductions in preventable ED visits for non-Hispanic African American (incidence rate ratio (IRR) = 0.81; 95% CI, 0.70-0.94) and Hispanic (IRR = 0.72; 95% CI, 0.60-0.87) enrollees relative to their White counterparts [16].

The distinct structural trend reversal identified in 2020 underscores a severe intersectional vulnerability. Within this non-expansion landscape, the onset of the pandemic temporally coincided with acute macroeconomic disruptions and labor market instability. Rather than demonstrating direct causality, these state-level macro-trends parallel the anticipated effects of systemic shocks in regions lacking comprehensive safety nets, such as expanded Medicaid. The data suggest that pre-existing vulnerabilities within the Hispanic female cohort were disproportionately exposed during this period of concurrent economic and public health instability. Jason et al. demonstrated that older Hispanic adults experienced significantly higher rates of job loss and greater financial hardships associated with COVID-19 compared to their Non-Hispanic White counterparts [17].

Beyond the severe reversal observed in Hispanic women, the segmented trend analysis suggests a broader gendered impact of the macroeconomic shock. While all female cohorts demonstrated statistically significant improvements in healthcare access prior to 2020, none maintained a significant trajectory of improvement post-2020. This aligns directly with the findings of Zamarro and Prados, who noted that working mothers assumed a disproportionate burden of caregiving responsibilities during the health crisis, which was associated with a reduction in working hours and a higher probability of transitioning out of employment [18]. However, our data highlight that the Hispanic female cohort was uniquely vulnerable within this gendered phenomenon, experiencing a complete and abrupt trend reversal (+1.28 AAC) rather than mere stagnation.

The identification of this structural break in 2020 at an ecological level suggests that cultural resilience may have critical limits when faced with external economic shocks. These findings align conceptually with previous Bayesian evidence from individual-level survey data in Latin American migrants, which has also suggested an erosion of the "healthy immigrant paradox" under conditions of high social vulnerability [19]. While the current ecological design precludes direct individual-level inferences, the consistency across different populations and methodologies reinforces the hypothesis that financial toxicity acts as a potent structural correlate of health advantage loss within Hispanic communities.

This study has several limitations inherent to its design. First, the ecological nature of the data utilizes state-level aggregated estimates, which preclude the inference of individual-level causal risks and make the findings susceptible to the ecological fallacy. Observed trends represent population-level shifts and should not be interpreted as direct evidence of individual health behaviors or outcomes. Second, critical unmeasured confounders-such as specific employment sectors, immigration status, and ethnic subgroup heterogeneity (e.g., country of origin)-were not adjusted for in this aggregated dataset, which may mask internal variations within the Hispanic cohort. Third, the BRFSS relies on self-reported data, subject to recall and social desirability biases. Importantly, the COVID-19 pandemic introduced potential non-sampling errors; modifications in BRFSS data collection and weighting procedures during the 2020-2022 period could have influenced prevalence estimates independently of socioeconomic changes. Finally, the repeated cross-sectional nature of the surveillance system allows for the identification of temporal associations but prevents definitive causal conclusions regarding the impact of specific economic shocks or policy decisions.

## Conclusions

This ecological analysis demonstrates the persistence of severe, structurally ingrained disparities in cost-related healthcare access in Florida, with Hispanic women bearing the most profound burden. Bayesian paired samples analysis provides very strong evidence of a macro-level intersectional disparity, revealing a stark 13.02-percentage-point mean paired gap compared to non-Hispanic White men. Furthermore, segmented trend analysis indicates that while access barriers for Hispanic women were steadily improving prior to 2020, the onset of the COVID-19 pandemic was associated with a pronounced structural break, abruptly halting the progress observed in the preceding decade. These findings highlight a critical intersectional vulnerability to macroeconomic shocks at the population level. Addressing this enduring inequity requires targeted state-level public health interventions and economic safety nets designed to protect vulnerable demographics during systemic crises. Finally, future research utilizing individual-level microdata remains essential to definitively disentangle these ecological disparities from underlying socioeconomic deprivation.
